# Effects of Ballistocardiogram Peak Detection Jitters on the Quality of Heart Rate Variability Features: A Simulation-Based Case Study in the Context of Sleep Staging

**DOI:** 10.3390/s23052693

**Published:** 2023-03-01

**Authors:** Ahmad Suliman, Md Rakibul Mowla, Alaleh Alivar, Charles Carlson, Punit Prakash, Balasubramaniam Natarajan, Steve Warren, David E. Thompson

**Affiliations:** Mike Wiegers Department of Electrical & Computer Engineering, Kansas State University, Manhattan, KS 66506, USA

**Keywords:** ballistocardiogram, Bayes error, classifier, electrocardiogram, heartbeat interval, k-nearest neighbor, R-J interval, R-R interval, support vector machine

## Abstract

Heart rate variability (HRV) features support several clinical applications, including sleep staging, and ballistocardiograms (BCGs) can be used to unobtrusively estimate these features. Electrocardiography is the traditional clinical standard for HRV estimation, but BCGs and electrocardiograms (ECGs) yield different estimates for heartbeat intervals (HBIs), leading to differences in calculated HRV parameters. This study examines the viability of using BCG-based HRV features for sleep staging by quantifying the impact of these timing differences on the resulting parameters of interest. We introduced a range of synthetic time offsets to simulate the differences between BCG- and ECG-based heartbeat intervals, and the resulting HRV features are used to perform sleep staging. Subsequently, we draw a relationship between the mean absolute error in HBIs and the resulting sleep-staging performances. We also extend our previous work in heartbeat interval identification algorithms to demonstrate that our simulated timing jitters are close representatives of errors between heartbeat interval measurements. This work indicates that BCG-based sleep staging can produce accuracies comparable to ECG-based techniques such that at an HBI error range of up to 60 ms, the sleep-scoring error could increase from 17% to 25% based on one of the scenarios we examined.

## 1. Introduction

Heartbeat intervals (HBIs) vary over time, and that variance can be quantified as a heart rate variability (HRV) feature, a quantity usually based on HBIs determined from R-R intervals in electrocardiograms (ECGs) [1,2,3,4,5,6,7,8,9]. Such HRV features support many health-related applications, including long-term health monitoring [10,11], sleep staging [1,12,13,14,15], and sleep quality assessment [16,17].

Electrocardiography is the gold-standard technique for HRV parameter measurement in clinical settings and, more recently, short-term, home-health monitoring environments, given the advent of wearable devices. For long-term health monitoring, the wires and electrodes required for electrocardiography introduce significant challenges.

**Figure 1:**
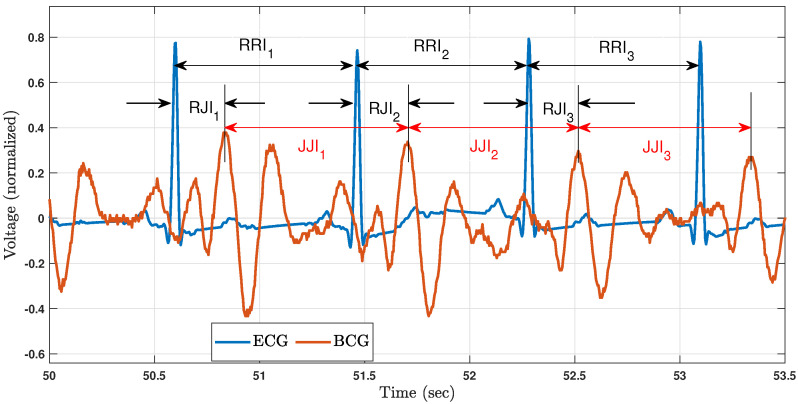
Heartbeat interval estimates from simultaneous ECG and BCG recordings. An R–R interval (RRI) represents the time between consecutive R peaks—the prominent peaks of an ECG that correspond to the onset of ventricular depolarization events. Similarly, a J–J interval (JJI) represents the time between consecutive BCG J peaks, where J peaks are the prominent peaks of a BCG, believed to be generated by blood ejection events at the aortic artery. Variations in RJIs due to physiology are also indicated in this figure, labeled as RJI${}_{n}$. Although RJI1, RJI2, and RJI3 are visually similar, they are numerically different. The signal amplitudes are normalized relative to their respective ranges across the entirety of each recording.

A ballistocardiogram (BCG), which is a signal derived from tissue micromovements caused by circulatory dynamics in the body, is an alternative source for HRV parameter estimation. A BCG-based measurement is preferable, in some settings, to an ECG-based measurement because a BCG does not require sensors to be attached to the body. However, the HBIs (and corresponding HRV features) obtained from a BCG are numerically different from those obtained from an ECG [2,3,4,5,6,7,8,9]. We refer to the differences between BCG- and ECG-based HBI and HRV values as HBI and HRV errors, respectively. This terminology reflects the fact that ECG-based HBI and HRV parameters are considered gold standards in this arena. Figure 1 illustrates several aspects of the ECG and BCG waveforms over a few cardiac cycles, demonstrating how even simultaneously recorded ECGs and BCGs can lead to different HBI estimates.

As the figure indicates, underlying timing differences exist. However, the importance of those differences is application-specific. We have chosen to focus on sleep staging. Quantifying the effects of HBI error on sleep staging accuracy is important, in part because it affects algorithm selection and development. While a few studies have used BCG-based HRV metrics for sleep staging [18,19,20], the effects of HBI error on the resulting outcomes seem to have been overlooked.

**Figure 2:**
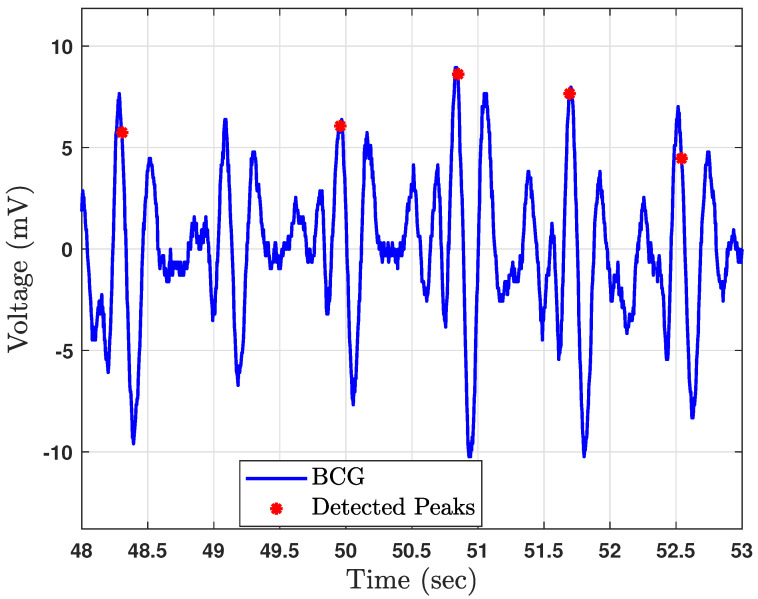
An example of perturbations in J–peak time estimates due to peak detection algorithm performance.

Note that HBI/HRV errors are due to several sources, e.g., physiology, sensing modality, and peak detection algorithm. We focus on those errors stemming from the BCG peak detection process. As observed in our previous works [21,22], peak detection methods introduce perturbations in the temporal locations of detected heartbeats. Missed and false peaks also contribute to HBI errors, but the focus of this research is the HBI error due to peak-detection-based timing jitter. Figure 2 illustrates a sample segment of BCG data where a detected heartbeat does not always align with the actual beat location. Importantly, such a mismatch is not always consistent; the error varies between heartbeats. This variability is due to the unpredictable nature of the error sources.

Another source of differentiation between ECG- and BCG-based HBIs is the variable time between ventricular depolarization and aortic blood ejection. The R peak of the ECG originates at the beginning of ventricular depolarization, whereas the J peak of the BCG is time aligned with blood ejection. Consequently, the R to J-peak interval (RJI) is not constant from beat to beat (see Figure 1). This RJI variability is a physiological component of the HBI error whenever a BCG is used, and it will be addressed in this work.

Based on our review, no published dataset incorporates simultaneous ECG and BCG recordings along with time-aligned expert sleep scores. However, the National Sleep Research Resource (NSRR) database [23,24,25,26] contains ECG and expert sleep scores, lacking only the BCG. Here, we simulate the missing BCG in order to allow an initial investigation of the feasibility of BCG-based sleep staging.

To that end, this research empirically investigates the effects of HBI errors on HRV feature quality and sleep staging accuracy. We use the results of sleep-stage classification and its sensitivity to HBI errors to demonstrate the effects of HBI error on sleep-scoring performance.

This work presents three primary contributions to the literature, (i) this is a novel, systematic investigation into the contribution of HBI error in sleep-scoring performance. The framework may be extended to other application-specific investigations. (ii) As part of this investigation, we have developed an estimated relationship between HBI error and sleep staging accuracy. This may serve as an assessment tool for other researchers developing sleep-scoring systems based on cardiac information before conducting human sleep studies. (iii) Finally, we believe this to be the first five-class automated sleep scoring using only HRV data. This study outlines an SVM-based sleep-scoring performance using HRV features only from a clean ECG dataset with well-labeled sleep scores. This establishes a reference for subsequent sleep-scoring studies.

## 2. Related Work

Both ECGs and BCGs have been used to extract HRV features for sleep staging. As indicated in Table 1, the performance metrics for the affiliated classification algorithms are roughly comparable. The column “Stages Classified” identifies the labeling schemes employed by the different studies. SWS stands for slow wave sleep, and N-SWS stands for non-SWS. Likewise, REM stands for rapid eye movement sleep, and N-REM stands for non-REM sleep.

Higher accuracies, of up to 89%, are also reported from other studies, as noted in [27]. However, those studies included other signals for sleep staging, e.g., respiratory effort data [28,29], in addition to ECGs.

**Table 1:** Classification performance metrics based on HRV features acquired from ECG and BCG signals.

Signal Used	Stages Classified	Acc. (%)	Sen. (%)	N	Cohen’s Kappa	Ref.
ECG	SWS vs. N-SWS	90	69	45	0.56	[27]
BCG + Actigraphy	SWS vs. N-SWS	93	81	4	0.62	[19]
ECG	REM vs. N-REM	87	87	25	0.61	[30]
BCG + movement	REM vs. N-REM	80	N/A	18	0.43	[20]

While BCG-based HRV features appear to yield comparable sleep staging results when employed in lieu of ECG-based HRV features (e.g., as laid out in Table 1), BCG-based sleep staging is not yet widely used. Therefore, the effects of any differences in BCG versus ECG-based HRV estimates are not well understood, where discrepancies stem from HBI errors as determined from JJI versus RRI.

A few studies have addressed such errors. For example, [20,31,32] described errors in HRV features due to HBI errors, and [6,17] reported correlation coefficients between BCG- and ECG-based HRV features. However, these papers do not discuss the effects of HBI errors on sleep staging accuracy. One study [33] did compare sleep stages determined with a partially BCG-based system [34] to polysomnogram-based sleep scores as determined by a polysomnographic technician. However, the impacts of HBI errors on the sleep staging accuracy cannot be calculated for this study since BCGs were not the only signals used for sleep staging.

Based on the review of the available literature, it appears that no formal investigation of the effects of HBI differences (as determined from ECG versus BCG signals) has been performed in the context of sleep stage determination. Sleep-based studies that employ BCG-based HRV features seem to be limited to [14,17,18,19,20,32,35,36,37]. Therefore, it seems worthwhile to investigate the effects of ECG- versus BCG-based HRV features on sleep-scoring performance results, particularly in light of the promise that BCG-based systems hold for unobtrusive sleep monitoring. Such an analysis can help set a performance target for BCG-based heartbeat detection algorithms, which must deal with natural beat-to-beat changes in signal morphology that are not as prevalent in ECGs.

Note that this effort parallels current research with photoplethysmograms (PPGs) as employed for sleep stage determination. Similar to a BCG, a PPG acquired from a finger or a wrist offers a remote indication of “pulse rate” that is different from “heart rate” as determined by an ECG, though we speak of each type of signal as providing HRV features. When acquiring a BCG or a PPG, the physical distance (and, therefore, the arterial layout) between the sensor and the heart affects the signal morphology, and beat-to-beat changes in signal shape are more pronounced in BCGs and PPGs when compared to ECGs. Additionally, the smoother, more oscillatory nature of each of these signals, when compared to ECGs with their distinct R waves, can make accurate heartbeat identification more of a challenge. Nonetheless, since light-based sensors used to acquire PPGs are less intrusive than electrodes used to acquire ECGs, recent efforts have investigated the benefits of PPGs to estimate HRV features in the context of sleep staging. For example, [38] compared PPG- and ECG-derived HR and HRV values, reporting good agreement based on the resulting correlation coefficients. However, other recent studies have indicated that using PPG-derived HR and HRV features can lead to a decrease in sleep staging performance [39].

## 3. Methods

### 3.1. Data Description

NSRR datasets from 50 participants were included in this study. Sleep scores identified for those 50 participants served as ground truth values for our sleep stage assessments. In order to investigate sleep stage scoring sensitivity to ECG-BCG timing differences, we introduced synthetic timing jitter to the ECG-based HBIs to mimic the effects of RJI variations.

**Figure 3:**
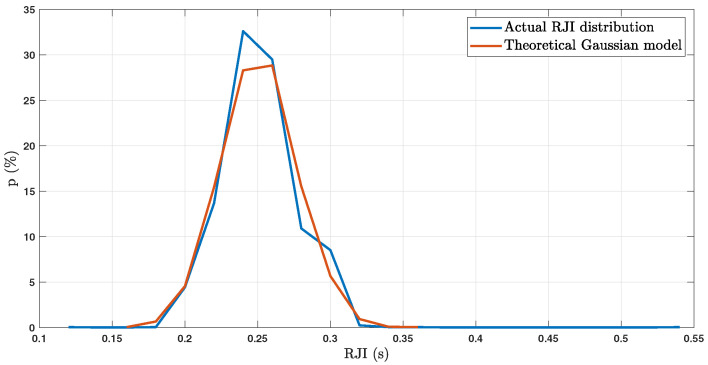
RJI data and a theoretical Gaussian fit based on mean and standard deviation of the RJI data.

Timing error can come from a combination of many sources, including BCG sensing modality, peak detection algorithm, and participant physiology. If these random sources sum together, the resulting error should be Gaussian by the Central Limit Theorem. In addition, a Gaussian model is a reasonable fit for our measured timing jitter in another study (described in Section 3.5, below). Figure 3 depicts an overlay of the observed RJI distribution (blue line) and that of a theoretical Gaussian model (red line). For this histogram, we removed data from one participant because their BCG was collected from a different location than all other participants in the original study.

For the jitters discussed in this paper, we used random time offsets drawn from a zero-mean Gaussian distribution because HBI calculations (and thus HRV calculations) are insensitive to a mean time offset.

**Figure 4:**
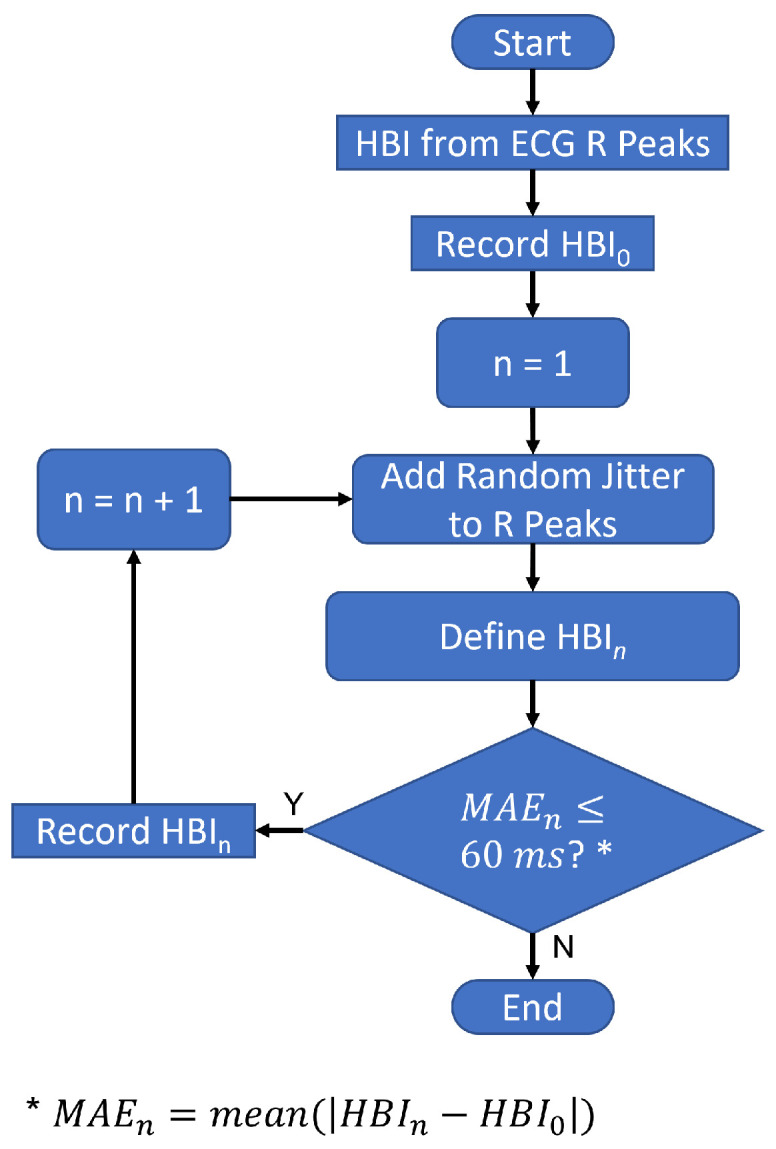
The process of adding jitter to the R-peak locations and recording the resulting RRIs.

ECG R peaks provided ground truth heartbeat times. We denote an HBI obtained from these R peaks as HBI_0_, where the zero subscript signifies zero added error. Then we obtained HBIs from the artificially perturbed R-peak locations. We denote HBIs obtained from these R peaks with timing jitter as HBI${}_{n}$, where *n* corresponds to an *n*th-level of error contamination. Thus, HBI${}_{n}$ represents an HBI calculated from an R-peak stream with an *n*th-level error. We increased the standard deviation of these perturbations until we reached a mean absolute error (MAE) between HBI${}_{n}$ and HBI${}_{0}$ of 60 ms. We specified 60 ms as a maximum based on the findings in [22], where HBI errors due to the worst-performing method were less than 60 ms for most of the participants. This process took 97 iterations, thus leading to *n* = 97 levels of synthetic HBI error. Figure 4 illustrates the process of obtaining HBIs with simulated errors starting with ECG R-peak locations provided in each dataset. The process was repeated 50 times to cover all of the datasets included in the study.

We refer to the MAE between each HBI${}_{n}$ and HBI${}_{0}$ as MAE${}_{n}$ on an individual level, where HBI MAE is an overall metric. For example, MAE${}_{97}$, the MAE between HBI${}_{97}$ and HBI${}_{0}$, is approximately 60 ms. MAE${}_{n}$ serves as the independent variable for this study.

### 3.2. HRV Features

We calculated frequency- and time-domain HRV features per 30-s epoch for HBI${}_{0}$ and for each HBI${}_{n}$ time series, using five-minute-wide sliding windows with 90% overlap [40]. As described in Section 3.1, we had 97 levels of synthetic errors in addition to the original times, so 98 sets of HRV features were obtained.

#### 3.2.1. Frequency-Domain Features

Low-frequency (LF) and high-frequency (HF) powers were calculated using Lomb–Scargle normalized periodograms [41,42] due to the unevenly sampled nature of all HBI time series. The LF and HF frequency ranges correspond to 0.04–0.15 Hz and 0.15–0.4 Hz, respectively [15]. In addition, the ratio of LF to HF powers (LFHF) and the one-dimensional median-filtered version of the ratio (MedFiltLFHF) were employed as frequency-domain features.

#### 3.2.2. Time-Domain Features

The mean of the HBIs (denoted by HR as in [40]) and its standard deviation (SDNN) in each window were used as time domain features.

### 3.3. Sleep Labels

The NSRR database provides sleep stage labels of Wake, S1, S2, S3, S4 (rarely), and REM [23,24,25,26]. The label S4, which is not a standard level, was very rare among these participants. Therefore, those labels were merged with S3. In addition, sleep stages shorter than four epochs were merged into their previous stage. In most of the cases, such behavior was observed as a glitch where the stages before and after the glitch were the same. The average sleep duration among these participants was 8.43 hours with a standard deviation of +/− 0.5 h. The average time spent in each sleep stage for these participants is summarized in Table 2. Two scenarios were tested: (i) micro-labeling, where all sleep stage labels were provided as class labels, as available from the NSRR database, and (ii) macro-labeling, where non-REM sleep stages were merged into a single stage and assigned a single label resulting in the three stages of REM, N-REM, and Wake, similar to what was performed in [20,30] (see Table 1).

### 3.4. Effects of HBI Error on HRV Feature Quality

We used Bayes error and classification error to quantify the effects of HBI error on HRV feature quality (see the sections below for the calculation methods used to determine these errors). Classification error was obtained using the support vector machine (SVM) classifier. SVM is a binary classifier that provides a class probability $\hat{y}$ of a sample instance using the following equation [43]:

(1) $$\begin{array}{c} \hat{y}\left(x\right)={\hat{w}}_{0}+\sum_{i=1}^{N}\alpha_{i}k\left(x_{i},x\right)\;\mathrm{Where},\;\alpha_{i}=\lambda_{i}y_{i},\;\lambda \;\mathrm{is}\;\mathrm{the}\;\ell_{1}\;\mathrm{regularization}\;\mathrm{term}. \end{array}$$

SVM finds the optimal values for ${\hat{w}}_{0}$ and α by maximizing the minimum distance between the two separating hyperplanes. Given that SVM only finds the decision boundary between two discrete class instances, SVM can not be used directly to solve multi-class problems. However, using one-vs.-all or one-vs.-one heuristic methods, we can solve multi-class classification tasks with SVMs. SVM is a well-established classifier with relatively compact representation of the training data and good test computation requirements. In addition, the SVM classifier is efficient in higher dimensional space and works well for a fewer number of samples than data dimensions. Therefore, we chose to use SVM here. In this study, we used the one-vs.-one strategy to implement SVM-based multi-class problems since the one-vs.-one approach creates more balanced binary datasets than one-vs.-all. To implement the strategy mentioned above, we used MATLAB’s templateSVM.m function with the Gaussian kernel, and to train the model, we used fitcecoc.m. A 10-fold cross-validation approach was adopted for this purpose. All HRV feature sets obtained from HBI${}_{0}$ to HBI${}_{97}$ were examined individually to obtain Bayes and classification errors corresponding to each HBI MAE.

Two scenarios were evaluated based on the micro- and macro-labeling schemes identified in Section 3.3. In addition, two tests were performed in each scenario. In the first test, all HRV features were used to estimate Bayes and classification errors. In the second test, individual features were used to estimate Bayes and classification errors. The second test was performed to investigate the sensitivity of each HRV feature to HBI error.

#### 3.4.1. Bayes Error Test

We used a non-parametric method to estimate the Bayes error. Parametric Bayes error estimation requires knowledge of class distribution parameters and prior probabilities, whereas non-parametric methods can be used in the absence of this information. A non-parametric method for an L-class classification problem is provided in [44], which relies on k-nearest neighbor (kNN) classification. The kNN classifier uses a predetermined distance metric to find the closest *k* training instances for a given test instance, and then it classifies the test instance based on the majority vote of the training labels for these “neighbors”. Given an L-class problem with a sufficiently large training dataset, the Bayes error bounds can be calculated using Equation (2) [45]:

(2) $$\begin{array}{c} \frac{L-1}{L}\left(1-\sqrt{1-\frac{L}{L-1}E_{kNN}}\right)\leq E_{bayes}\leq E_{kNN} \end{array}$$

Here $E_{kNN}$ is the *kNN* classifier error. Again, both micro- and macro-labeling test scenarios were addressed. Within each scenario, individual and multiple HRV features were considered.

#### 3.4.2. Classification Error Test

For this test, classification error was obtained using the HRV feature sets. An overall classifier error pattern in response to HBI error is obtained as well. This error pattern is in the form of the slope of the classifier error curves due to each HRV feature within the *n*th HRV feature set. This slope represents the change in classification error percentage per millisecond change in the HBI MAE of the underlying HBI${}_{n}$. The slope was obtained from a linear regression of the classifier error against the HBI MAE${}_{n}$, the independent variable.

### 3.5. Brain and Body Sensing Laboratory Data

While we have argued that simulated BCG data are better for our analyses, they do have one shortcoming. ECG-BCG timing differences include a physiological component—the variation between R-peak time and physical blood ejection—which cannot be estimated or subtracted from the BCG signal alone. This variability provides a lower bound for the timing error for BCG systems and must be estimated from a dataset that has both ECGs and BCGs. Therefore, we used simultaneous ECG-BCG data recorded in the Kansas State University Brain and Body Sensing Laboratory [21,22] to estimate this lower bound.

This archive included data from 30 healthy volunteers: 14 males (ages 30.9 ± 6.3 years) and 16 females (ages 46.0 ± 18.5 years). These data were initially collected for the purpose of algorithm comparison, as outlined in [22]. We excluded three volunteers for this analysis, as their data lacked simultaneous ECG recordings. One hundred pairs of consecutive ECG- and BCG-based heartbeats were used for each subject, leading to a total of 2700 heartbeat pairs.

### 3.6. Comparing the Present Study with Previous Work: Simulated Versus Laboratory-Based ECG-BCG Timing Errors

We previously compared peak-detection algorithms for BCG signals [21,22], focusing on the time difference between each ground truth and detected J peak, an error we will denote here as $e_{p}$. The ‘*p*’ indicates that the source of the error is the peak detection algorithm. However, the error discussed in this present work is the total time difference between each ground-truth ECG R peak and the respective detected J peak. This total error then reflects not only the timing jitter from peak detection but also physiology and platform effects. The total error, which we denote here as $e_{T}$, is not directly comparable to $e_{p}$.

Therefore, we must augment our previously reported $e_{p}$ values with platform and physiology effects, i.e., R to ground-truth J peak intervals (RGIs), before comparing them with $e_{T}$ values obtained by adding synthetic timing jitter to the ECG peaks of the NSRR dataset employed in the current study. This may be performed by considering the following:

(3) $$\begin{array}{c} e_{T}=RGI+e_{p} \end{array}$$

This equality stems from the fact that each peak is detected at a time offset, which is the sum of the platform offset, the physiology offset, and the peak detection offset. Taking the difference of adjacent detected peak times leads directly to the previous equation. The use of the triangle inequality allows the comparison of the two quantities:

(4) $$\begin{array}{c} |e_{T}\left|\leq |RGI|+\right|e_{p}| \end{array}$$

Therefore, we will supplement the mean $e_{p}$ values previously reported to account for platform and physiology effects, which are reflected in the R-to-J interval, or the difference between the times of arrival of the R and J peaks. While the mean RJI is unimportant because HRV features are insensitive to mean offsets, the difference between each RJI and the mean RJI is a source of error. Thus, we have calculated the total error by adding $e_{p}$ and the mean absolute “jitter” between each RJI and the mean RJI. That is, the parameter |RGI| in Equation (4), for each participant, *i*, is defined as

(5) $$\begin{array}{c} {\left|RGI\right|}_{i}=\left|RGI_{i}-\frac{\sum_{j=1}^{M_{i}}RGI_{j,i}}{M_{i}}\right| \end{array}$$

where $M_{i}=100$ is the number of R-J pairs from each participant, *i*, from our previous work [22] for whom we had collected time-aligned ECGs and BCGs.

This method will lead to a conservative upper bound for the total system error. The true error might be lower since peak detection jitter can offset platform and physiology effects for at least some beats.

## 4. Results

### 4.1. Classifier Baseline Performance

**Figure 5:**
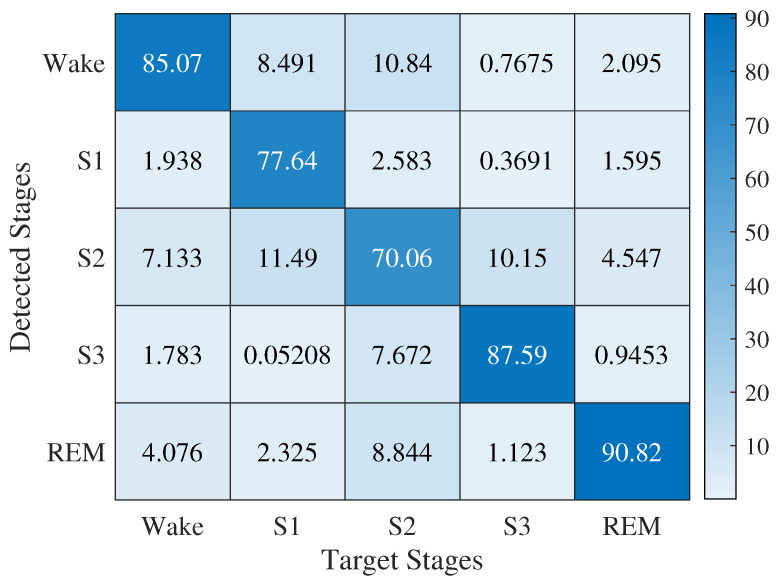
SVM classification baseline performance due to all HRV features for micro-labeled sleep stages.

Figure 5 is a confusion matrix based on classification performed on the HRV features when no timing jitters were introduced to the RRI data. For this confusion matrix, SVM was used. This confusion matrix is based on 33 participants out of 50 after discarding results that would contain zeros in any detection column.

### 4.2. HBI Error Effects

Table 3 summarizes the SVM classification errors, $E_{0}$ and $E_{max}$, for the extreme cases of HBI${}_{0}$ and HBI${}_{97}$, respectively (We did consult the kNN classifier-based results, and they showed overall worse performance than SVM, though it was slightly less sensitive to the HRV errors added in the study. For brevity, we have included figures that relate only to the SVM classification method).

**Figure 6:**
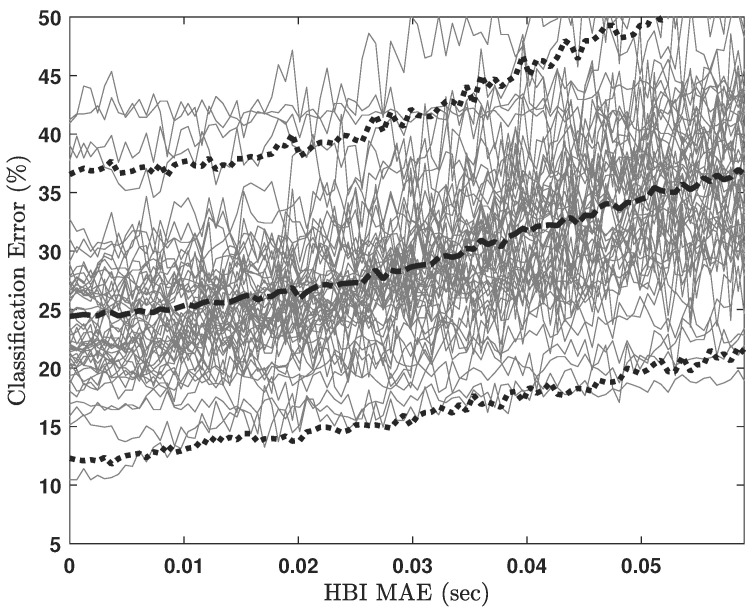
SVM classification error due to all HRV features for micro-labeled sleep stages. Light gray lines represent classification errors for each participant. The dashed, bold black line is the ensemble average, and the dotted bold lines show mean +/− 1.96 standard deviations.

**Figure 7:**
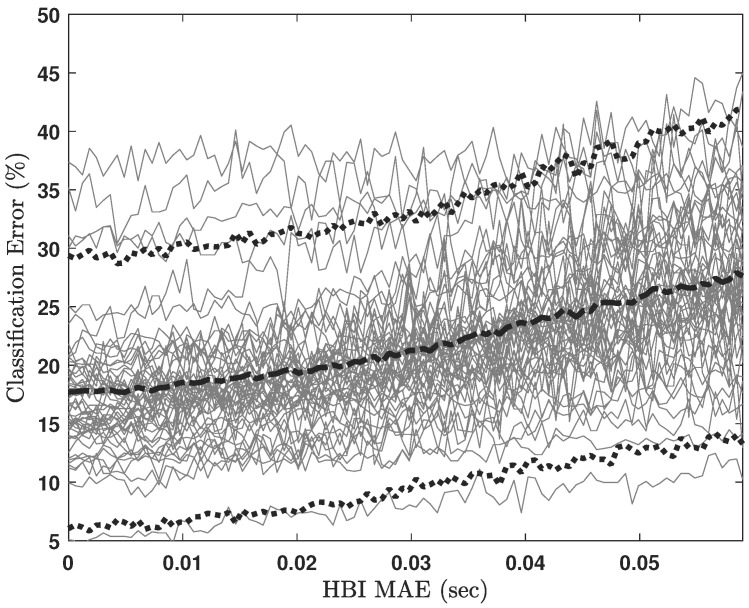
SVM classification error due to all HRV features for macro-labeled sleep stages. Light gray lines represent classification errors for each participant. The dashed, bold black line is the ensemble average, and the dotted bold lines show mean +/− 1.96 standard deviations.

Figure 6 illustrates the SVM classification error as a function of HBI MAE for the micro-labeled sleep stages, and Figure 7 illustrates the SVM classification error as a function of HBI MAE for the macro-labeled sleep stages. In both cases, all HRV features were provided to the classifier. Classification errors for each participant are graphed in light gray lines, whereas the dashed, bold black line in each figure is the ensemble average. As expected, increasing HBI MAE led to increasing sleep staging error. At the same time, the classification error seems to remain low (≤20% in the case of micro-labeled sleep stages) for roughly half of the simulated MAE${}_{n}$ values.

Table 4 summarizes the sensitivity of the HRV features to changes in HBI MAE. The numbers in this table are the slopes described in Section 3.4.2 averaged across participants. The sensitivities for HF, LFHF, and MedFiltLFHF are very close. Note that the units are in percentage points (pp) per second of change in MAE, so the change in performance is on the order of a few percentage points over the 60 ms range investigated in this study.

### 4.3. BCG-Based HBI Error Limit

Table 5 lists some statistical metrics for |RGI|, $|e_{p}|$, and their sum. The numbers are based on 2700 heartbeats (100 beats from each participant, with a total of 27 participants), and the $|e_{p}|$’s are obtained from J peaks detected using an optimal BCG peak detection approach by Brüser [5] as identified in our previous papers [21,22].

As explained in Section 3.6, the sum of |RGI| and $|e_{p}|$ is an upper bound for total mean error between BCG-based and ECG-based HBIs. Thus, this quantity is comparable to the HBI MAE parameter utilized for the horizontal axes in Figure 6 and Figure 7. From Table 5, the mean for this quantity is approximately 16 ms. If we locate 16 ms on the curves in Figure 6 and Figure 7, it corresponds to up to 26% and 18% sleep classification errors for micro- and macro-labeling schemes, respectively. Table 6 compares the macro-labeling errors projected from our analyses to state-of-the-art sleep-scoring errors compiled from Table 1.

## 5. Discussion

We demonstrated that HBI errors due to timing jitters follow a Gaussian trend owing to the inherent variability across R to J peak intervals in the context of BCG. This trend is the basis of our simulation-based results with the following implications.

### 5.1. Timing Jitter in Heartbeat Detection

The best-case micro-labeling classification error on this dataset, with no added timing jitter, is 24%. While any timing jitter decreases performance, all practical systems have some timing jitter. From the literature (see Table 1), a range of errors from 10% to 20% can be inferred, though all of those studies used macro-labeling, which makes it hard to put our micro-labeling performance in perspective. Macro-labeling performs better. The classification error for macro-labeling would be as low as 17% in the best case (see Table 3).

Ideally, the classification error would be lower. Yet detection of sleep staging from cardiac data is not trivial—fast heart rates could result from nightmares, waking events, and several other sources. Traditional polysomnography includes EEG electrodes as well as other non-cardiac data to achieve lower classification error. Here, we have investigated the use of BCG-only signals, which could allow sleep staging without wires or sensors on the participant, thus potentially allowing multi-month or multi-year studies, which are simply not practical with the existing technology.

### 5.2. HRV Feature Sensitivity to HBI MAE

The slopes of the error curves provide insight into each HRV feature’s sensitivity to HBI MAE. From Table 4, we see that HR is the feature least sensitive to HBI MAE in both the macro- and micro-labeling scenarios. The slope is noticeably smaller than it is for other features over the examined range of HBI MAEs, particularly for the macro-labeling scenario. This finding is consistent with our understanding of theoretical sensitivity. HR is calculated using windowed averages of HBIs, which smooths out the effects of introduced HBI perturbations. In fact, a perturbation to a heartbeat time inside of a window does not substantively change the calculated HR unless the perturbation affects one of the outside two heartbeats or if that perturbation moves a heartbeat into or out of the window. On the other hand, HF and LFHF are the features most sensitive to HBI MAE in macro- and micro-labeling scenarios, respectively. Again, this matches intuition—both of these features include frequency-domain content that is more sensitive to perturbations inside of the window. As expected, these sensitivities are lower in the case of macro-labeling since the number of labels is reduced, and thus, classification performance is improved.

### 5.3. Notes Regarding the Use of BCG-Based HBIs

It must be noted that when comparing an HBI estimation algorithm’s performance with the HBI MAE values obtained here, the reference for both should be the R peaks of a simultaneous ECG. For other references, some calibration should first be considered. As mentioned in the Methods section, for instance, the HBI errors based on BCG ground truth peaks were calibrated to include the effects of RJI variability and the measurement platform.

### 5.4. Projected HBI-Based Sleep-Scoring Performance

As noted in Table 6, the projected sleep macro-labeling performance based on our proposed HBI error limit compares well with other recent results. Note that the 7% sleep labeling error reported in [19] is based on both BCG and actigraphy (acceleration) signals and is not, therefore, directly comparable.

### 5.5. Limitations and Future Work

This study relies on the results of simulated HBI errors. The BCG recording system introduced in [46,47,48,49,50] and the BCG peak detection method selected in [21,22] puts us in a position to estimate reliable HBIs and HRV features. This is because this work offers insight into the best- and worst-case HBI error scenarios, which will put the BCG-based HBIs and their related HRVs in perspective for us. As indicated earlier, there is no available dataset of simultaneous ECG, BCG, and sleep scores. Such a dataset would have enabled us to assess the other effects of peak detection algorithm artifacts, e.g., missed and false detection events. Nonetheless, this work can be a stepping stone toward further studies in that direction. Eventually, we hope to conduct sleep studies where we record polysomnograms and BCGs simultaneously on a larger population.

## 6. Conclusions

We examined the effects of HBI errors in the context of one BCG application: sleep staging. For that purpose, we introduced different levels of synthetic errors to ECG-based HBIs in order to simulate BCG-based HBIs. HRV features were then calculated from these altered HBIs and used for sleep staging. We used these HRV features with the SVM classification algorithm and one-vs.-one strategy to enable a multi-class classification. In addition, different sleep stage labeling and HRV feature combination scenarios were tested. Our results indicate a clear trend toward decreasing sleep staging accuracy with increasing timing jitter. The sensitivity varies substantially between HRV feature type. We cannot recommend a universal amount of acceptable jitter because the cost of staging errors will depend on the end application. Nevertheless, the results indicate that timing jitters as small as 20 ms demonstrate a measurable impact on the overall performance of HRV-based sleep labeling algorithms.

We examined classification performance sensitivity to HBI errors while providing individual features to each classifier. As expected, classification based on heart rate was the least sensitive to this type of error.

Based on the HBI error range proposed in this work, the feasibility of BCG-based HBI estimation for sleep stage classification was assessed.

## Figures and Tables

**Table 2:** Average percentage of sleep duration in each stage for the study cohort.

Wake	S1	S2	S3	REM
30.0%	2.6%	41.1%	11.6%	14.7%

**Table 3:** SVM classification errors averaged over 50 participants for HBI${}_{0}$ and HBI${}_{97}$.

Labeling	$E_{0}$ (%)	$E_{\max}$ (%)
Micro-Labeling	24	36
Macro-Labeling	17	27

**Table 4:** Sensitivity of feature quality to HBI error in terms of classifier mean error slopes (units: percent error per second of HBI MAE).

Features	HR	SDNN	LF	HF	LFHF	MedFiltLFHF	Label
Slopes (%/s)	4.37	27.10	19.66	58.74	30.32	31.78	Micro
	4.43	27.26	19.44	59.02	30.37	32.01	Macro

**Table 5:** Statistical metrics for |RGI|, $|e_{p}|$, and their sum.

	Mean (ms)	Median (ms)	Min (ms)	Max (ms)
|RGI|	7.16	6.09	0.94	44.44
$|e_{p}|$	8.77	8.26	1.27	23.60
$\left|RGI|\;+\;\right|e_{p}|$	15.93	14.35	2.21	68.04

**Table 6:** State-of-the-art macro-labeling performance with respect to projected performance based on the HBI error limit.

Signal Used	Stages Classified	Ref.	100-Acc. (%)	Projected Error (%)
ECG	SWS vs. N-SWS	[27]	10	18
BCG + Actigraphy	SWS vs. N-SWS	[19]	7	
ECG	REM vs. N-REM	[30]	13	
BCG + movement	REM vs. N-REM	[20]	20	

## Data Availability

Not applicable.
